# Sympathetic Activation and Baroreflex Function during Intradialytic Hypertensive Episodes

**DOI:** 10.1371/journal.pone.0036943

**Published:** 2012-05-22

**Authors:** Dvora Rubinger, Rebecca Backenroth, Dan Sapoznikov

**Affiliations:** Nephrology and Hypertension Services, Department of Medicine, Hadassah University Medical Center, Jerusalem, Israel; Université de Montréal, Canada

## Abstract

**Background:**

The mechanisms of intradialytic increases in blood pressure are not well defined. The present study was undertaken to assess the role of autonomic nervous system activation during intradialytic hypertensive episodes.

**Methodology/Principal Findings:**

Continuous interbeat intervals (IBI) and systolic blood pressure (SBP) were monitored during hemodialysis in 108 chronic patients. Intradialytic hypertensive episodes defined as a period of at least 10 mmHg increase in SBP between the beginning and the end of a dialysis session or hypertension resistant to ultrafiltration occurring during or immediately after the dialysis procedure, were detected in 62 out of 113 hemodialysis sessions. SBP variability, IBI variability and baroreceptor sensitivity (BRS) in the low (LF) and high (HF) frequency ranges were assessed using the complex demodulation technique (CDM). Intradialytic hypertensive episodes were associated with an *increased* (n = 45) or *decreased* (n = 17) heart rate. The maximal blood pressure was similar in both groups. In patients with *increased* heart rate the increase in blood pressure was associated with marked increases in SBP and IBI variability, with suppressed BRS indices and enhanced sympatho-vagal balance. In contrast, in those with *decreased* heart rate, there were no significant changes in the above parameters. End-of- dialysis blood pressure in all sessions associated with hypertensive episode was significantly higher than in those without such episodes. In logistic regression analysis, predialysis BRS in the low frequency range was found to be the main predictor of intradialytic hypertension.

**Conclusion/Significance:**

Our data point to sympathetic overactivity with feed-forward blood pressure enhancement as an important mechanism of intradialytic hypertension in a significant proportion of patients. The triggers of increased sympathetic activity during hemodialysis remain to be determined. Intradialytic hypertensive episodes are associated with higher end-of- dialysis blood pressure, suggesting that intradialytic hypertension may play a role in generation of interdialytic hypertension.

## Introduction

During hemodialysis treatment, blood pressure usually decreases with ultrafiltration and weight loss. In a significant proportion of patients, however, the blood pressure increases during or immediately after hemodialysis. Intradialytic hypertension has been recognized for many years, and it is believed to occur in at least 8–15% of patients [1]. There is no standard definition for intradialytic hypertension; some of the most common accepted criteria reviewed by Chazot and Jean [2] include a 15 mmHg increase of mean arterial pressure between the start and the end of a dialysis session [3], hypertension resistant to ultrafiltration occurring during or immediately after the dialysis procedure [4], or at least a 10 mmHg increase in the systolic blood pressure from pre-to post-dialysis [5]. Recent data have shown that intradialytic hypertension is associated with increased mortality and morbidity [5], [6].

Several mechanisms are believed to lead to intradialytic increases in blood pressure. A positive sodium balance, resulting in extracellular fluid overload and hypertension in dialysis patients, is thought by many investigators to be the main cause of intradialytic increases in blood pressure [2], [7]. Other popular hypotheses link intradialytic hypertension to variations in potassium or ionized calcium concentrations [8], [9], antihypertensive drug removal during hemodialysis [10], hemoconcentration [11], recombinant erythropoietin administration, stimulation of the renin-angiotensin system during ultrafiltration [12], and to hemodynamic changes including increased cardiac output and vasoconstriction [13]. The latter is believed to be caused by endothelial dysfunction [14] and/or with increased endothelin secretion and altered nitric oxide/endothelin balance [13], [15].

Sympathetic overactivity, believed to be generated by neuro-hormonal mechanisms arising within the diseased kidneys, is considered an important mechanism of hypertension in patients with chronic renal insufficiency [16]–[18]. Sympathetic nervous system activation during hemodialysis was proposed to be an important factor in the pathogenesis of intradialytic hypertension, via an increase in cardiac output and/or an increase in peripheral resistance. This hypothesis was supported by initial reports on increased plasma levels or increased turnover of cathecholamines in uremic patients and in experimental models of renal insufficiency [19]–[21]; no such changes, however, were found during hemodialysis treatment [22], [23]. Furthermore, the baseline and intradialytic plasma levels of chatecholamines were reported to be similar in intradialytic hypertension-prone and in sex- matched control hemodialysis patients [15].

Sympathetic activity is best detected using microneurography (efferent post-ganglionic muscle sympathetic nerve activity, MSNA) and regional norepinephrine spillover technique [24]. Previous studies have shown that the basal MSNA firing was double in hypertensive hemodialysis patients as compared with age-matched healthy controls with normal blood pressure. Most studies, however, were performed in patients with residual renal function or in hemodialysis patients on a non-dialysis day [16], [17], [25]. To our knowledge, no such studies were performed in patients with intradialytic hypertension.

Since direct methods are not available or applicable in complex patients, sympathetic activity is assessed in many studies by non-invasive techniques. Heart rate variability (HRV), blood pressure variability (BPV) and baroreflex function (baroreceptor sensitivity, BRS) are the most frequently used methods to assess autonomic nervous system function and cardiovascular variability, a measure of the integrated control mechanisms of circulation [26]. In clinical settings, these measurements are usually computed from continuous beat to-beat recordings of heart rate and blood pressure oscillations [26]–[28]. Thus, the behavior of heart rate is considered an indirect index of adrenergic cardiovascular drive [29]. Spectral analysis of the heart rate oscillations allows the identification of low frequency (LF) component, believed to result from neural mechanisms related to sympathetic and vagal outflows, and a high frequency component (HF), related to respiration; the LF/HF ratio is frequently used as an index of sympatho-vagal interaction on the autonomic control of heart rate [26]–[28], [30], [31]. In humans, alterations of the low frequency band of blood pressure variability (LF SBP) were shown to be affected by sympathetic modulation of vascular tone as well as by myogenic vascular function [26]–[28], [32].This relationship was well documented by studies measuring muscle sympathetic nerve activity in healthy volunteers, in whom sympathetic stimulation and nerve activity were associated with predominance of LF oscillations of blood pressure and of LF interbeat intervals [33], [34]. In the recent years, in many studies, “spontaneous” BRS indices are also determined from continuous beat to beat surveys of blood pressure and heart rate, without pharmacological or external interventions. Both spontaneous and laboratory BRS indices correlate negatively with BPV and positively with HRV [26]–[28].

Complex demodulation is a newer method for assessing time dependent changes in signals around a specific frequency. The technique of complex demodulation (CDM) reveals instantaneous dynamic changes in heart rate and blood pressure that are not revealed by the standard spectral methods [35]. Therefore CDM may be particularly suitable to assess dynamic rapid changes in the autonomic nervous system during dialysis.

The present study was undertaken: 1. to detect increases in blood pressure during hemodialysis, and, 2. to assess the role of autonomic nervous system function during intradialytic hypertensive episodes. This is the first report of cardiovascular variability indices determined before and during intradialytic hypertensive episodes using the CDM methodology.

## Methods

Continuous beat to beat blood pressure recordings were obtained during 113 routine midweek dialysis sessions in 108 chronic patients on maintenance hemodialysis. Patients with chronic atrial fibrillation or frequent ventricular premature beats, with debilitating illness and frequent hospital admissions, with permanent pacemakers or with severely decreased radial pulses following multiple vascular access surgical interventions were excluded from the study. Antihypertensive drugs were stopped 12–14 hr before the dialysis session. The study was approved by Hadassah Medical Organization Ethics Committee for Clinical Research. The patients signed an informed consent form before taking part in the study.

Demographic, clinical and laboratory data of all patients are shown in Tables 1 and 2. The patients were dialyzed 3.5 to 4 hours three to four times weekly, using the Gambro AK 200 delivery system and polyamide high flux dialyzers. The mean urea reduction rate (%URR) in all patients was 65–75%. Data on dialysate sodium concentration are given in Table 2; dialysate chloride, bicarbonate, potassium, dextrose and calcium concentrations were 108, 35, 2, 12 and 1.5 mmol/l, respectively.

**Table 1:** Demographic and clinical data of all patients and of patients without and with hypertensive episodes.

	All Patients (n = 108)	Without hypertension (n = 51)	With hypertension (n = 57)	p^a^
Age (years)^b^	58 (15)	56 (16)	60 (15)	0.216
Males/females	69/39	32/19	37/20	0.815
Dialysis vintage (years)^b^	1.90 (3.02)	2.21 (3.51)	1.61 (2.49)	0.304
History of hypertension [n (%)]	98 (90.7)	45 (88.2)	53 (93.0)	0.396
Diabetes mellitus [n (%)]	38 (35.2)	18 (35.3)	20 (35.1)	0.982
History of ischemic heart disease [n (%)]	62 (57.4)	25 (49.0)	37 (64.9)	0.095
LV systolic dysfunction [n (%)]^c^	28 (27.2)*	12 (23.5)	16 (30.8)	0.409
Diastolic dysfunction [n (%)]	37 (40.7)**	15 (34.1)	22 (46.8)	0.217
History of hyperlipidemia [n (%)]	79 (73.1)	37 (72.5)	42 (73.7)	0.894
History of frequent hypotensive episodes [n (%)]	53 (49.1)	23 (45.1)	30 (52.6)	0.434
Antihypertensive medication [n (%)]	80 (74.1)	36 (70.6)	44 (77.2)	0.578
Nitrates [n (%)]	20 (18.5)	5 (9.8)	15 (26.3)	0.027
Calcium blocking agents [n (%)]	40 (37.0)	15 (29.4)	25 (43.9)	0.121
Vasodilators^d^ [n (%)]	20 (18.5)	3 (5.9)	17 (29.8)	0.001
ACE inhibitors [n (%)]	24 (22.2)	10 (19.6)	14 (24.6)	0.536
Angiotensin receptor blockers [n (%)]	10 (9.3)	4 (7.8)	6 (10.5)	0.631
Beta-blockers [n (%)]	55 (50.9)	25 (49.0)	30 (52.6)	0.708
Plasma Creatinine (µmol/l)^b^^,^ e	728 (200)	757 (208)	702 (190)	0.154
Plasma calcium (mmol/l)^b^^, ^ e	2.22 (0.19)	2.24 (0.20)	2.21 (0.19)	0.434
Plasma phosphate (mmol/l)^b^^, ^ e	1.55 (0.36)	1.56 (0.37)	1.54 (0.36)	0.848
Hb (g/dl)^b^^, ^ e	11.1 (1.2)	11.1 (1.1)	11.2 (1.3)	0.751
Plasma albumin (g/l)^b^^, ^ e	38.0 (4.2)	38.7 (3.8)	37.4 (4.4)	0.112
PTH (pmol/l)^b^^, ^ e	49 (41)	51 (39)	48 (43)	0.684
CRP (mg/dl)^b^^, ^ e	3.36 (4.31)	2.85 (3.67)	3.82 ( 4.80)	0.254

a With vs. without hypertension.

b Mean (SD).

c Left ventricular ejection fraction (LVEF)<40%.

d Doxasozine or hydralazine.

e Predialysis values.

* Assessed in 103 patients, 52 with hypertensive episodes.

** Assessed in 91 patients, 47 with hypertensive episodes.

ACE, angiotensin-converting enzyme; CRP, C-reactive protein; Hb, hemoglobin; LV, left ventricle; PTH, parathyroid hormone.

**Table 2:** Dialysis data of all patients and of patients without and with hypertensive episodes.

		All Patients (n = 108)	Without hypertension (n = 51)	With hypertension (n = 57)	p^a^
Predialysis SBP (mmHg)*		134 (25)	135 (27)	133 (23)	0.694
Intradialytic weight loss (% of body weight)^b^		27.5 (15.3)**	28.1 (15.7)	26.9 (15.1)	0.714
Ultrafiltration rate (ml/min)^b^		9.29 (4.62)**	9.56 (4.67)	9.10 ( 4.57)	0.630
Dialysate Na concentration [n (%)]	<140 mmol/l	2 (1.9)	1 (2.0)	1 (1.8)	0.663
	140 mmol/l	98 (90.7)	45 (88.2)	53 (93.0)	
	>140 mmol/l	8 (7.4)	5 (9.8)	3 (5.2)	
Predialysis plasma Na (mmol/l)^b^		139 (3)	139 (3)	139 (3)	0.340
Postdialysis plasma Na (mmol/l)^b^		140 (3)^c^	141 (4)^c^	140 (3)^d^	0.245
Gradient between dialysate and plasma Na (mmol/l)^b^		1.20 (3.52)	1.00 (3.70)	1.39 (3.38)	0.572
Increase in plasma Na after dialysis (mmol/l)^b^		1.37 (2.98)	1.45 (3.02)	1.30 (2.97)	0.792
Predialysis plasma K (mmol/l)^b^		4.97 (0.65)	5.10 (0.76)	4.85 (0.65)	0.065
Postdialysis plasma K (mmol/l)^b^		3.54 (0.43)^c^	3.63 (0.37)^c^	3.45 (0.47)^c^	0.030
Increase in plasma K after dialysis (mmol/l)^b^		1.43 (0.62)	1.47 (0.62)	1.40 (0.62)	0.537
Predialysis plasma osmolality (mosm/KgH_2_O)^b^		318 (12)	319(10)	316 (12)	0.187
Postdialysis plasma osmolality (mosm/KgH_2_O)^b^		305 (8)^c^	307 (8)^c^	303 (8)^c^	0.054

* Data given as median and interquartile range.

** Data available in 97 patients (53 with hypertensive episodes).

a With vs. without hypertension.

b Mean (SD).

p vs. predialysis values:

c <0.001;

d 0.002.

The ultrafiltration rate during dialysis sessions never exceeded 1 liter/hour and was adjusted according to the presumed dry weight. The temperature of the dialysate was kept constant at 36°C.

All patients were treated with recombinant erythropoietin and phosphate binders (calcium carbonate, lanthanum carbonate and sevelamer hydrochloride) as needed.

### Blood pressure signal acquisition

Blood pressure and inter-beat intervals were measured noninvasively by Finometer 1.10 (FMS, Finapres Medical Systems BV, Arnhem, The Netherlands). Continuous beat to beat recording of the finger arterial pressure waveform was performed as previously described [36] using the volume-clamp method of Penaz [37] and the Physiocal criteria of Wesseling [38]. In the volume-clamp method the diameter of the finger artery under the inflated cuff is kept constant. Changes in the arterial diameter, detected by a photo-plethysmographic method cause, via fast servo controller, pressure changes in an inflatable air bladder within the finger cuff. The Physiocal algorithm adjusts the correct diameter at which the finger artery is clamped. The transmural pressure is therefore zero all the time i.e., the finger cuff pressure equals the intra-arterial pressure. The finger cuff was wrapped on the middle phalanx of the middle finger. A height correction unit was attached to correct for finger height changes during the recording. An upper arm cuff unit was also attached and used to calibrate the systolic pressure for calculating the reconstructed brachial pressure from the finger pressure.

**Figure 1:**
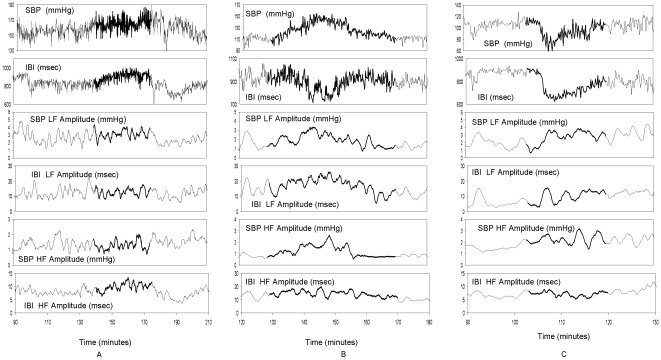
Representative tracings of continuous recordings of SBP and IBI oscillations and variability during intradialytic hypertension episodes with *decreased* (A) or *increased* heart rate (B). Tracings of a patient with marked intradialytic hypotension are depicted for comparison (C). The episodes associated with sudden changes in blood pressure are shown by the bolder parts of the tracings. LF, low frequency; HF, high frequency; IBI, interbeat interval.

The data from Finometer were downloaded into a PC and analyzed by the BeatScope Software (TNO TPD Biomedical Instrumentation Amsterdam, The Netherlands) and our specifically designed programs. The BeatScope software performs beat to beat analysis of the finger arterial pressure and uses filtering and level correction to calculate reconstructed brachial pressures from finger pressures. The resulted data files containing beat to beat values of hemodynamic variables such as inter-beat intervals (IBI), systolic (SBP), diastolic (DBP) and mean blood pressures (MBP), were used for further processing. Data files included beat to beat values for the whole duration of hemodialysis. SBP and IBI signals were resampled at 1 Hz to obtain equidistant time series [35]. Aliasing was negligible as the Nyquist frequency is 0.5 Hz for this sampling rate and it was found to be above the frequency content of SBP and IBI signals. The recordings were scanned for hypertensive episodes. The *hypertensive episodes* were defined as: 1. a period of at least 10 mmHg increase in the systolic blood pressure (SBP) between the start and the end of a dialysis session, or, 2. hypertension occurring after significant ultrafiltration was achieved, i.e. during the second to the fourth hour of dialysis or immediately after the dialysis procedure [2], [6]. During hemodialysis, blood pressure was also measured at 20 minute intervals using a digital oscillometric sphygmomanometer. During hypertensive episodes, blood pressure was double-checked by two different nurses or technicians every 5 minutes. The intradialytic episodes (n = 62) of increased blood pressure were further divided according to IBI changes into: a) *episodes with decreased IBI*, when the increase of at SBP was associated with a parallel increase in heart rate (n = 45) and, b) *episodes with increased IBI*, when the SBP increase was accompanied a decrease in heart rate (n = 17). Representative tracings of hypertensive episode associated with *increased* or *decreased* rate are depicted in Figure 1, left and mid panels. An episode with intradialytic hypotension and increased heart rate is depicted for comparison in the right panel.

### Complex demodulation

Complex demodulation is a nonlinear time domain method of time series analysis. It is suited to the investigation of nonstationary oscillations and reveals instantaneous dynamic changes that are not revealed by the power spectrum methods. The analysis used in the present study was based on technique of Hayano et al [35]. In this analysis, signal X (t) is assumed to contain a frequency component which changes slowly around a specified center frequency, while Y(t) is the complex signal after shifting the frequency band around the frequency of interest to zero (see **Methods S1**).

Complex demodulation of SBP and IBI signals was performed at two frequency bands. The central frequencies (f_0_) were 0.09 Hz for the low frequency (LF) and 0.30 Hz for the high frequency (HF) band. The result was then filtered by a low-pass 16th degree Butterworth filter with cut-off frequencies of 0.05 Hz and 0.16 Hz for the low and high frequencies range respectively. The signals were then smoothed by a 201 point least-square moving polynomial. Complex demodulation baroreflex (BRS) time series was computed as the ratio of the IBI and SBP time series instantaneous values in the two frequency ranges.

The amplitudes of SBP, IBI and BRS changes in the low (around center frequency of 0.09 Hz) and high (around center frequency of 0.30 Hz) frequency ranges were followed during the whole dialysis session. Five minute averages of the LF and HF SBP, LF and HF IBI, LF IBI/HF IBI ratio (representative of sympatho-vagal balance) and BRS indices were calculated the following manually detected periods: 1) the first 20 minutes (beginning), 2) the last 20 minutes (end) of dialysis, 3) 20 minutes before, and, 4) during hypertensive episodes.

### Statistics

Demographic and clinical categorical variables were compared by *chi*-square test. Age and laboratory data (mean and standard deviation) were compared using Student's *t* test. Blood pressure, inter-beat interval and baroreceptor variables are given as medians and inter-quartile ranges.

Wilcoxon signed-ranks test was used to compare paired differences at the beginning and the end of dialysis and to assess differences between the periods before and during hypertensive episodes. Mann-Whitney U-test was used to compare between sessions without and with hypertension, and between hypertensive episodes with increased and decreased heart rate.

Correlations of intradialytic changes in SBP with CDM indices and between different CDM variables were performed by Pearson correlation and linear regression analysis.

### Logistic regression analysis

Logistic regression analysis (stepwise forward conditional procedure) was performed to select determinants of intradialytic hypertension. Age, gender, hemodialysis vintage (years), the presence of diabetes mellitus, history of hypertension, hyperlipidemia , ischemic heart disease and of intradialytic hypotensive episodes, administration of antihypertensive drugs, hypertensive medication, biochemical data and predialysis SBP, IBI and CDM measurements (LF IBI, HF IBI, LF SBP, HF SBP, LF BRS and HF BRS) were all included in the analysis.

### Definitions of clinical variables

Ischemic heart disease was defined by: documentation of prior myocardial infarction or of coronary interventions, the presence of significantly abnormal Q waves on a 12-lead electrocardiogram, symptomatic angina pectoris or a thallium perfusion scan suggestive of myocardial ischemia. Left ventricular systolic dysfunction was defined as decreased left ventricular ejection fraction (LVEF) to less than 40% on echocardiography. Doppler interrogation of the mitral valve, pulmonary vein and mitral annulus was used to assess diastolic function. Severe diastolic dysfunction was defined as :a) pseudo-normalization of E (early filling wave) to A (late filling wave) ratio, suggesting an elevated left ventricular diastolic pressure, or, b) a restrictive pattern, suggesting reduced compliance, i.e. grade 2–4 diastolic dysfunction by echocardiographic criteria [39]. Patients with high triglyceride or/and high LDL cholesterol levels were considered to be hyperlipidemic.

## Results

### Clinical and laboratory data

One hundred and eight patients were examined during 113 hemodialysis sessions. Hypertensive episodes (n = 62) were detected in 57 patients. The timing of the occurrence of the hypertensive episode was different for each patient. Ten episodes occurred in the 1^st^, 22 in the 2^nd and^ 24 in the 3^rd^ hour, and 6 towards the end of hemodialysis session. The duration (mean ± SD) of the hypertensive episodes was 27±16 min (range 5–80 min). While many patients with intradialytic increases in blood pressure episodes complained of dizziness, palpitations or weakness, none developed syncope or loss of consciousness.

Patient clinical and biochemical data are listed in Table 1. Pre-and post dialysis SBP and other data related to the hemodialysis procedure are listed in Table 2. Age, sex distribution and dialysis vintage were similar in patients without and with intradialytic hypertensive episodes (Table 1). Diabetic nephropathy was the primary renal disease in 15 (29.4%) and in 18 (31.6%) patients without and with hypertensive episodes, respectively. Other causes of end stage renal failure in patients without and with intradialytic hypertension were glomerulonephritis (10 and 11), interstitial nephritis (5 and 5), familial nephropathy in 6 (6 and 3), nephrosclerosis and ischemic nephropathy (3 and 8), autosomal dominant polycystic kidney disease (1 and 3) and miscellaneous (11 and 9). A residual renal function was present in 44 patients, 17 without (mean creatinine clearance 5.87(4.12) ml/min) and 27 with intradialytic hypertension (mean creatinine clearance 5.29(3.65) ml/min, pNS). A history of essential or secondary hypertension was in the medical background of 45 (88.2%) and 53 (93%) patients without and with intradialytic hypertensive episodes, respectively (p NS). While the prevalence of diabetes mellitus, hyperlipidemia and the dialysis vintage was similar in both groups, that of ischemic heart disease was slightly increased in patients with hypertensive episodes (p = 0.095) . Fifty three (49.1%) patients, 23 patients with and 30 without hypertensive episodes (p NS) had a history of frequent hypotensive episodes during dialysis. The prevalence of patients receiving antihypertensive drugs was comparable in both groups, but the use of vasodilators and nitrates was more frequent in patients with intradialytic hypertensive episodes. There were no significant differences between groups with regard to cardiac function, biochemical data, dialysis prescription, intradialytic weight loss or ultrafiltration rate. While the decrease in plasma K^+^ was similar in both groups, post dialysis K^+^ level was lower in patients with hypertensive episodes (Table 2).

### Effect of hemodialysis treatment on autonomic activity and on baroreceptor sensitivity in patients without and with intradialytic hypertensive episodes

Table 3 lists blood pressure, interbeat intervals and their variabilities, baroreflex sensitivity indices and LF IBI/HF IBI ratio during all screened hemodialysis sessions (n = 113), and sessions without (n = 51) and with (n = 62) hypertensive episodes. There was a significant decrease in SBP at the end of hemodialysis sessions without hypertensive episodes, while in those with hypertensive episodes, there were marked increases in SBP, DBP and MBP. A minimal decrease in LF SBP was noted, especially in session without hypertension, while HF SBP significantly increased at the end of dialysis in both groups. In both types of sessions, IBI was not significantly changed at the end of the dialysis. At the beginning of dialysis, LF BRS and HF BRS were higher in sessions associated with hypertensive episodes. Significant increases in LF IBI, HF IBI and LF BRS, and a decrease in LF IBI/HF IBI at the end of the treatment occurred only in the dialysis sessions without hypertension.

**Table 3:** Blood pressure variability, baroreflex sensitivity indices, interbeat intervals and low frequency/high frequency interbeat interval ratio at the beginning and at the end of hemodialysis*.

	All sessions (n = 113)**			Sessions without hypertension (n = 51)**			Sessions with hypertension (n = 62)**		
	Beginning	End	p	Beginning	End	p	Beginning	End	p
SBP (mmHg)	135 (31)	134 (36)	0.160	139 (42)	125 (34)	0.023	132 (30)	143 (36)^c^	0.001
DBP (mmHg)	67 (18)	73 (17)	0.001	69 (19)	71 (18)	0.056	67 (17)	76 (21)^d^	0.001
MBP(mmHg)	90 (20)	94 (22)	0.001	92 (25)	90 (16)	0.888	90 (17)	100 (17)^c^	0.001
IBI (msec)	827 (177)	862(216)	0.103	802 (196)	813(274)	0.606	850 (162)	878 (175)	0.089
LF SBP (mmHg)	1.92 (2.02)	1.90 (0.87)	0.726	1.98 (0.93)	1.95 (1.00)	0.051	1.92 (0.64)	1.88 (0.86)	0.188
HF SBP (mmHg)	1.40 (0.72)	1.69 (0.70)	0.001	1.54 (0.58)	1.89 (1.01)	0.003	1.21 (0.72)^b^	1.64 (0.52)	0.003
LF IBI (msec)	5.79 (4.74)	6.95 (6.38)	0.022	5.39 (4.49)	6.01 (6.80)	0.046	6.11 (4.92)	7.16 (5.49)	0.165
HF IBI (msec)	5.52 (3.28)	5.85 (4.07)	0.001	4.99 (2.45)	5.82 (5.32)	0.001	5.81 (3.39)	5.87 (2.77)	0.369
LF BRS (msec/mmHg)	3.48 (2.51)	3.60 (2.85)	0.003	3.03 (1.95)	3.21 (3.20)	0.001	3.62 (2.79)^a^	3.67 (2.67)	0.468
HF BRS (msec/mmHg)	3.87 (2.91)	3.69 (3.41)	0.783	3.29 (2.62)	3.42 (4.52)	0.376	4.39 (3.13)^b^	3.89 (2.54)	0.147
LF IBI/HF IBI	1.11 (0.59)	1.06 (0.50)	0.034	1.15 (0.64)	1.04 (0.58)	0.003	1.07 (0.57)	1.07 (0.60)	0.809

* Data given as median and interquartile range;

** Number of patients: all sessions-108; without hypertension-51; with hypertension-57. Beginning-the initial 20 minutes of the dialysis session; End-the last 20 minutes of the dialysis session. Comparison of sessions without and with hypotension-Beginning:

a p = 0.004;

b p = 0.008; End:

c p = 0.001;

d p = 0.035.

### Blood pressure and interbeat interval variability, baroreflex sensitivity and sympatho-vagal balance during intradialytic hypertensive episodes

Forty-five (72.5%) intradialytic hypertensive episodes (mean duration 24±14 min) in 41 patients were associated with an *increase* in heart rate; the heart rate *decreased* during 17 (27.5%) episodes in 16 patients (lasting 34±19 min, p NS).The timing of occurrence of hypertension during dialysis was similar in the two types of episodes. Five episodes were detected on repeated examinations; one patient was examined 3 times, and the remaining 3 patients were examined twice. Intradialytic hypertensive episodes with increased heart rate were detected during repeated examinations in 3 patients; in one remaining patient intradialytic increase in blood pressure with decreased heart rate was detected during two separate sessions.

**Figure 2:**
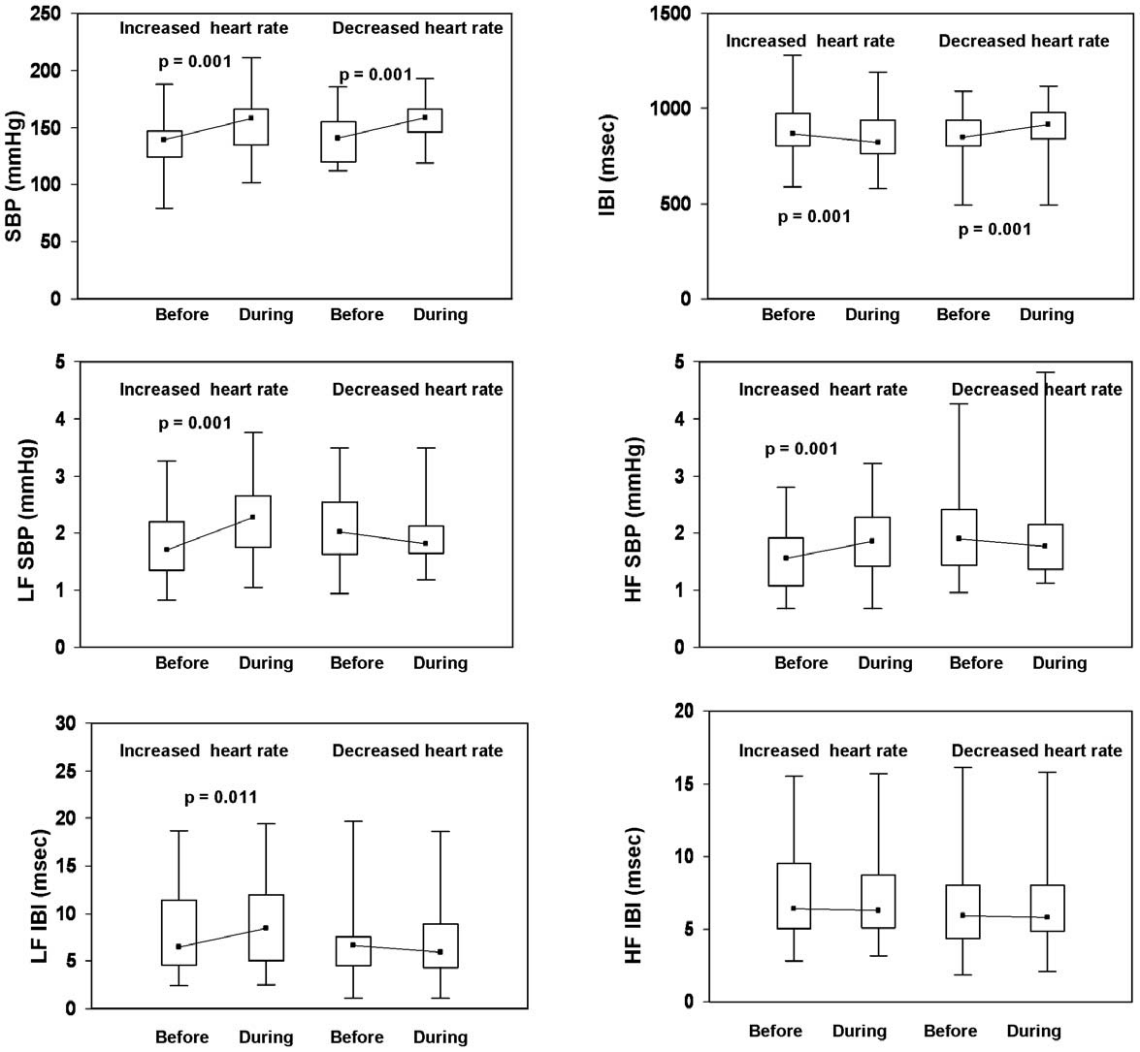
SBP and interbeat interval and their variability indices before and during intradialytic hypertensive episodes associated with *increased* (n = 45) or *decreased* (n = 17) heart rate. Data are presented as box plots. The box stretches from the 25^th^ to the 75^th^ percentile; the median is shown as a small black square in the box. The range (the upper and the lower extreme values) is indicated by whiskers. HF, high frequency; LF, low frequency, IBI, interbeat interval.

**Figure 3:**
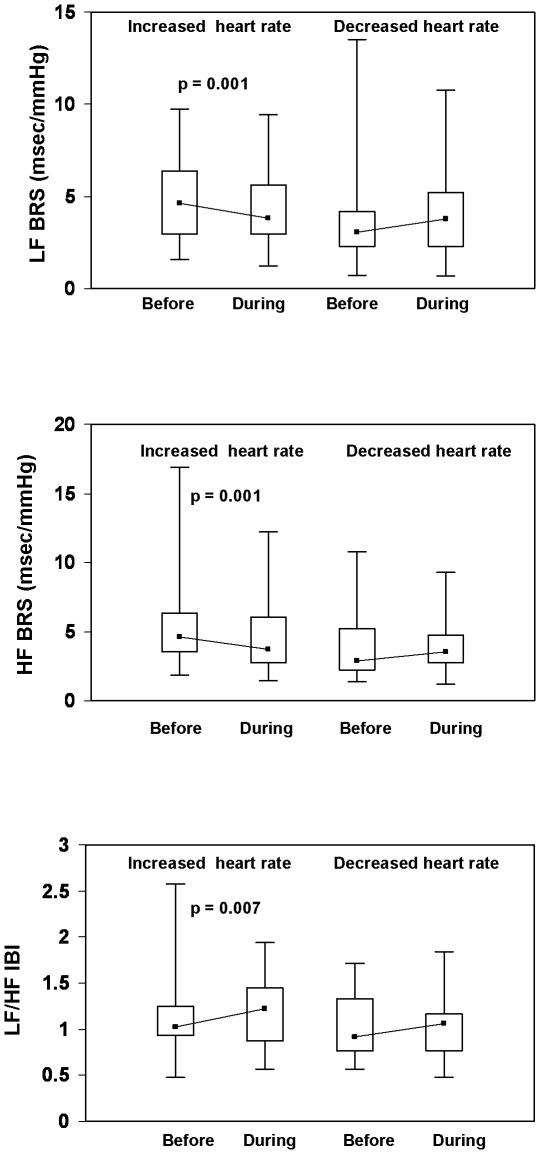
BRS indices and LF IBI/HF IBI ratio before and during intradialytic hypertensive episodes associated with *increased* (n = 45) or *decreased* (n = 17) heart rate. Data are presented as box plots. The box stretches from the 25^th^ to the 75^th^ percentile; the median is shown as a small black square in the box. The range (the upper and the lower extreme values) is indicated by whiskers. BRS, baroreceptor sensitivity; HF, high frequency; LF, low frequency, IBI, interbeat interval.

During hypertensive episodes, the magnitude of the increase in blood pressure was similar in both subgroups. Maximal SBP, DBP and MBP [median (interquartile range)] were 158 (30), 80 (24) and 104 (19) mm Hg in patients with *increased* heart rate (p<0.001 vs. before) and 159 (25), 73 (17) and 102 (19) mm Hg in patients with *decreased* heart rate (p<0.001 vs. before). SBP, IBI and their variability before and during hypertensive episodes are depicted in Figure 2, while baroreceptor sensitivity indices and LF IBI/HF IBI during intradialytic hypertension are shown in Figure 3. At the initiation of hypertensive episodes (before), blood pressure and IBI were similar in all patients. LF SBP, HF SBP and LF IBI markedly increased during the hypertensive episodes in patients with *increased* heart rate (decreased IBI) , but did not significantly change in those in whom heart rate *decreased* (increased IBI, Figure 2). In *increased* heart rate episodes, the blood pressure elevation was associated with significantly diminished baroreflex indices (LF BRS and HF BRS) and enhanced LF/HF ratio (Figure 3). In *decreased* heart rate episodes, a tendency to increase in BRS indices was observed during hypertension; these changes, however, were not statistically significant.

**Figure 4:**
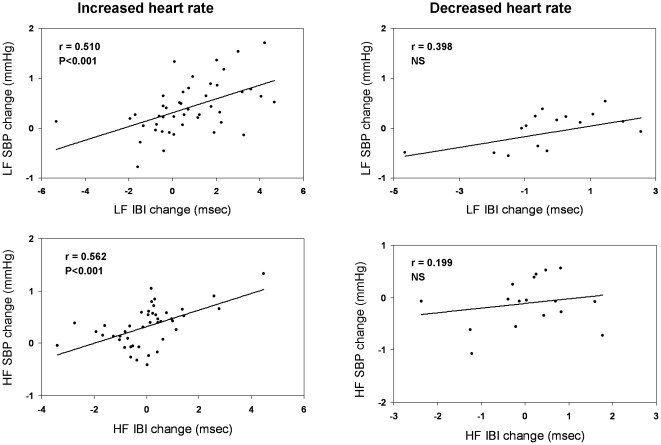
Correlations of changes in interbeat intervals with changes in SBP during intradialytic hypertensive episodes associated with *increased* (n = 45) or *decreased* (n = 17) heart rate. HF, high frequency; LF, low frequency, IBI, interbeat interval.

Significant correlations between changes in heart rate variability with changes in blood pressure variability in both LF (r = 0.526) and HF (r = 0.405) bands (p<0.001) were found during hypertensive episodes, but only in those with *increased* heart rate (Figure 4). We found no correlations between the blood pressure increases during hypertensive episodes with changes in heart rate variability or blood pressure variability, with changes in baroreflex indices or LF IBI/HF IBI ratio. Likewise, there were no correlations between blood pressure increases and weight loss, ultrafiltration rate, the dialysate to predialysis plasma Na concentration gradient or the decrease in plasma potassium and in plasma osmolality during dialysis.

Comparisons of patients experiencing intradialytic hypertension with *increased* or *decreased* heart rate showed that age, medical history and comorbidities, the number of years in dialysis, dialysis prescription and the biochemical profile were similar for the two subgroups (not shown). A higher proportion of women, 9 of 16, was noted, however, in the subgroup of patients with decreased heart rate as compared with 11 of 41 in patients with increased heart rate during hypertensive episodes (p = 0.036). In addition, in the former subgroup, 8 (50%) patients were treated with an angiotensin converting enzyme inhibitor as compared with 6 (14.6%) patients in the latter (p = 0.005). Pre-and post-dialysis SBP, DBP, IBI and CDM measurements were similar in patients with increased or decreased heart rate during intradialytic hypertension episodes.

### Predictors of intradialytic hypertension

Logistic regression analysis (stepwise forward conditional procedure) was performed to define independent predictors of intradialytic hypertension. Three different models were constructed including clinical, dialysis and laboratory data and CDM measurements (see **Methods**). The most significant predictors are listed in Table 4. Predialysis baroreflex sensitivity in the low frequency range (LF BRS) was found to be the most important predictor of intradialytic hypertension (1^st^ and 2^nd^ models, Odds Ratio 1.455 and 1.650, p = 0.004 and 0.001, respectively). Because of the strong correlation of baroreflex sensitivity with heart rate variability and the inverse correlation with blood pressure variability [28], these indices were analyzed in a different model (3^rd^ model). In this model, a higher predialysis LF IBI (Odds ratio 1.228, p = 0.006) and a lower LF SBP (Odds ratio 0.223, p = 0.001) were found to be significant predictors of hypertensive episodes.

**Table 4:** Predictors of intradialytic hypertension based on logistic regression analysis.

Variables	Predictors	Odds ratio	95% CI	b coefficient	p
**1^st^ Model** (Clinical data, LF BRS and HF BRS)	LF BRS	1.455	1.130–1.875	0.375	0.004
**2^nd^ Model** (Clinical and laboratory data, LF BRS and HF BRS)	Age	1.032	1.002–1.063	0.031	0.037
	Plasma albumin	0.875	0.781–0.979	−0.134	0.020
	LF BRS	1.650	1.222–2.228	0.501	0.001
**3^rd^ Model** (Clinical and laboratory data, LF IBI, HF IBI, LF SBP, HF SBP)	Albumin	0.892	0.801–0.993	−0.114	0.037
	LF IBI	1.228	1. 061–1.422	0.206	0.006
	LF SBP	0.223	0.089–0.561	−1.499	0.001

CI: confidence interval.

Variables in logistic regression models:

Clinical data: age, gender, dialysis vintage, history of intradialytic hypotension, history of hypertension, diabetes, ischemic heart disease, hyperlipidemia, anti- hypertensive medication, pre-dialysis SBP and IBI.

Laboratory data: pre-dialysis creatinine, calcium, phosphate, potassium, albumin, hemoglobin and PTH concentrations and CRP levels.

CDM measurements: pre-dialysis LF IBI, HF IBI, LF SBP, HF SBP, LF BRS and HF BRS.

Other significant predictors were lower serum albumin level (2^nd^ and 3^rd^ models, p = 0.020 and 0.037) and age (2^nd^ model, p = 0.037).

## Discussion

This study is the first, to our knowledge, to report on dynamic changes of SBP, IBI and baroreflex function during intradialytic hypertensive episodes. The principal new finding of this study is that a large proportion of hypertensive episodes are associated with increased heart rate, with marked increases in blood pressure variability (LF SBP and HF SBP), interbeat interval variability (LF IBI), decreased baroreceptor sensitivity (LF BRS and HF BRS) and enhanced sympatho-vagal balance (Figure 2 and 3).The above episodes, characterized by shortening of IBI intervals synchronous with SBP increase, are similar to those occurring during central autonomic activations [40]. These non- baroreceptor- related events are believed to be mediated by enhanced sympathetic outflow with feed-forward effects on blood pressure [41]. In contrast, during baroreceptor activation, increases or decreases in blood pressure are associated with feed-back mediated decreases or increases in heart rate (increased or decreased interbeat interval), respectively. In hemodialysis patients, baroreflex mechanisms were shown to be adequately activated during hypotensive episodes [36]. Our present findings suggest that sympathetic activation and feed–forward enhancement of blood pressure play an important role in the pathogenesis of intradialytic hypertension episodes.

### Sympathetic activation during intradialytic hypertensive episodes

Sympathetic overactivity associated with chronic hypertension was detected by MSNA in patients with early or advanced chronic kidney disease and on maintenance hemodialysis [17], [18], [25], [29], [42]. In one single study, performed during dialysis, the sympathetic firing was shown to increase during the hemodialysis procedure, ultrafiltration and decrease in blood pressure [18].

We have previously shown that in chronic dialysis patients, LF SBP was increased, while LF IBI and the baroreceptor sensitivity indices were markedly decreased as compared to normal individuals [43].These findings, similar to those reported in essential hypertension and congestive heart failure, were interpreted as evidence of background chronic sympathetic overactivity associated with end-stage kidney disease.

In the present study, the alterations noted during 45 (72.5%) of 62 intradialytic hypertensive episodes (Figures 2 and 3) are suggestive of acute bursts of sympathetic activity with feed forward enhancement of blood pressure. In the remaining 17 hypertensive episodes, similar increases in blood pressure were associated with decreased heart rate; no significant changes in interbeat interval variability, blood pressure variability or baroreflex indices were noted in these patients. One possible explanation for these findings may involve simultaneous co-activation of the parasympathetic system resulting in an elevated blood pressure without an increase in heart rate, as described in subjects under psychological stress [44]. The intradialytic elevation in blood pressure with no increase in heart rate might also be caused by mechanisms independent of autonomic nervous-system. Endothelial dysfunction was recently documented in patients with intradialytic hypertension [14], [45]; in one study intradialytic hypertension was independently associated with both endothelial dysfunction and increased arterial stiffness [45]. Accordingly, a primary functional defect within the arterial wall may lead under special circumstances to an exaggerated pressure response due to increased endothelin and decreased nitric oxide (NO) availability and increased vascular resistance [14], [15].

The mechanisms of acute intradialytic activation of the sympathetic nervous system are not well defined. Activation of RAS was suggested as a potential mechanism of both sympathetic stimulation and intradialytic hypertension. A recent study, however, did not show increases in plasma renin and aldosterone levels during hemodialysis in patients with intradialytic increases in blood pressure [15]. In our study, end-of dialysis plasma potassium level was lower in patients with hypertensive episodes. The lower plasma potassium in these patients could indeed have resulted from intradialytic activation of the renin-angiotensin system, leading to increased circulating angiotensin and aldosterone levels, with further activation of central nervous system mineralocorticoid and angiotensin type I receptors and enhanced sympathetic outflow [25], [46]. In hemodialysis patients, an acute decrease in serum potassium was shown to be associated with increased blood pressure, presumably mediated by arteriolar constriction via sympathetic stimulation [8].

Another proposed mechanism extensively investigated in chronic kidney disease links between renal ischemia and hypoxia, activation of mechano- and chemo-receptors, secretion of adenosine and sympathetic activation [42]. The relevance of this mechanism, however, for hemodialysis-associated hypertension remains to be determined.

Tonic arterial chemoreceptor activation was also shown to be involved in sympathetic activation in patients with advanced renal failure [47]. Hypoxemic events during hemodialysis, initiated by intrapulmonary (ventilation-perfusion mismatching) and/or extrapulmonary (ventilatory control) mechanisms [48] may elicit sympathetic activation [47]. Peripheral chemoreflex sensitivity is particularly enhanced in patients with obstructive sleep apnea, a common condition in chronic hemodialysis and frequently associated with hypertension. [49]. Studies in experimental animals have shown that prolonged blood flow reduction to carotid body enhances chemoreflex function and causes sympathetic activation [50]. Alterations of cerebral blood flow may occur in association with hemodynamic changes during hemodialysis [51].Taken together, these findings strongly support the hypothesis that sympathetic overactivity may be triggered by chemoreceptor dysregulation associated with fluid and electrolyte shifts during hemodialysis.

Other possible, non-mutually exclusive mechanism of intradialytic sympathetic activation may be related to a reduced vascular and hypothalamic availability of nitric oxide (NO) associated with alterations in sympathetic outflow and in vascular endothelial function [13], [15], [52]. Deficiency of the soluble monoamine oxidase renalase in end stage renal failure was also shown to be associated with increased sympathetic tone and with resistant hypertension [53]. The activation of the above mechanisms during intradialytic hypertensive episodes has yet to be explored.

Insufficient sodium removal during hemodialysis and volume excess were proposed and are still believed by many investigators to be the main causes of intradialytic and interdialytic hypertension [2], [7], [54], [55]. In experimental animals, excess salt may lead to hypertonic Na accumulation in skin, to endothelial activation and hypertension [56], [57]. A central effect of excessive salt intake may occur via enhancement of the excitability of sympatho-regulatory circuits in the rostral ventro-lateral medulla and it was hypothesized that even small changes in plasma sodium may affect the responsiveness of these neurons [57]. Salt balance may also affect sympathetic activity via modulation of renalase expression [53]. Dialysis salt loading may occur with increased dialysate Na concentration and/or with increased dialysate to predialysis plasma Na gradient [55]. Indeed, a mild but significant increase in plasma sodium concentration was noted after dialysis in all our patients (Table 2). Pre-and post dialysis plasma Na and the dialysate to predialysis Na concentration gradient, however, were similar in patients without and with hypertension (Table 2), and in hypertensive patients with increased as compared with those with decreased heart rate (see **Results**). Furthermore, these indices did not predict the occurrence of hypertensive episodes in the logistic regression models (Table 4). Thus, while a positive salt balance might be present in many of our patients, it could not explain the sudden increase in blood pressure or spontaneous bursts of sympathetic activity occurring during dialysis.

### Predictors of intradialytic hypertensive episodes

On logistic regression analysis, a higher predialysis BRS in the low frequency band (LF BRS) was found to be the main predictor of intradialytic hypertension (Table 4). Indeed, both LF BRS and HF BRS were higher at the beginning of dialysis sessions associated with intradialytic hypertension as compared with sessions without hypertensive episodes (Table 3). LF IBI was also higher, albeit not significantly at the beginning of hypertensive sessions. The increased *initial* BRS and LF IBI may suggest the presence of a more robust autonomic nervous system in patients with intradialytic hypertension and may be interpreted as resulting from the synergistic effect of enhanced sympathetic tone coupled with reflex vagal activation [58]. A recent paper underscores the dependency of the sympathetic baroreflex with the level of sympathetic activity at rest especially in young and old men and in postmenopausal women [59]. Extrapolation of these findings to our study, however, has to be done cautiously, in view of the different methodology and different study population. Genetic traits, including polymorphism of RAS genes [60] or of beta- adrenergic receptors [61] that affect autonomic activity and BRS were also not checked in the present study.

Comparisons of patients with and without hypertensive episodes (Table 1) shows a slightly higher ratio of ischemic heart disease and an increased proportion of patients treated with nitrates, calcium channel blockers and vasodilator drugs in the hypertensive group. These conditions, however, were reported to be most commonly associated with decreased or no significant changes in BRS [62]–[65]. The proportion of patients treated with beta blocker agents and with angiotensin receptors antagonists (ARB's) that may both increase BRS [66], [67] was similar in patients with and in those without hypertension. When compared with patients with increased heart rate, a higher proportion of those with decreased heart rate were treated with ACE inhibitors. These agents were shown to enhance heart rate variability and increased baroreflex sensitivity [68]. Even though antihypertensive medication was stopped in all patients at least 12 hr before the study, a partial effect of drugs on CDM measurements can not be excluded.

Other predictors of intradialytic hypertension in the present study were lower plasma albumin and age. These factors may be suggestive of the link between autonomic imbalance and chronic inflammation in elderly individuals and in patients with end stage renal disease on maintenance hemodialysis [69], [70]. The correlation of these markers with intradialytic hypertension has yet to be understood.

### Intradialytic hypertension may contribute to interdialytic hypertension

The second important finding of this study is the markedly increased end-of-dialysis blood pressure in patients with intradialytic hypertensive episode (Table 3) as compared with the remaining patients, in which end-of dialysis blood pressure decreased. This finding confirms previous observations from other studies [54], [71]; in one such study, intradialytic hypertension was associated with increased time-integrated blood pressure burden as measured by 44-hour ambulatory monitoring [71]. In our study, end-of dialysis blood pressure was similar in patients with hypertensive episodes with increased or decreased heart rate (not shown in tables). We did not find differences in ultrafiltration rates, intradialytic weight loss, pre-and post-dialysis plasma sodium concentration or dialysate sodium concentration between patients with and those without hypertensive episodes. Since blood volume monitoring techniques were not available, our patients' dry weight, however, was assessed by clinical criteria only. Thus, a residual volume and/or sodium excess in certain patients could not be ruled out, especially in the presence of a positive dialysate to plasma Na concentration gradient (Table 2). Chronic volume excess is thought to be the main cause of interdialytic hypertension. Within this regard our study corroborates the assumption that intradialytic hypertension may play a role in the generation of interdialytic hypertension [54].

### Limitations

One main shortcoming of our study is the use of noninvasive methodology of assessment of autonomic nervous function, since more direct techniques as MNSA or pharmacological challenge were not applicable to our patient population. The study patient population included many elderly individuals with multiple comorbidities. These patients however, are most characteristic of the present day general hemodialysis population. Likewise, because of the methodology limitations, patients with arrhythmia or pacemakers were excluded from the study. Furthermore, blood volume and hemodynamic parameters were not assessed in parallel with the CMD determinations.

### Perspectives

Our findings support the hypothesis that increased sympathetic activity mediates intradialytic hypertension. Intradialytic hypertensive episodes with elevated post-dialysis blood pressure may contribute to interdialytic hypertension. This association deserves further studies using hemodynamic monitoring, different dialysis protocols and pharmacological approaches. The prognostic impact of intradialytic sympathetic activation and hypertension on overall survival and cardiovascular morbidity of these patients remain to be further clarified.

## Supporting Information

Methods S1 (DOC)
