# Supplementary material for: Sympathetic Activation and Baroreflex Function during Intradialytic Hypertensive Episodes
Source: PLoS One. 2012 May 22;7(5):e36943. doi: 10.1371/journal.pone.0036943 (PMC3358286; doi:10.1371/journal.pone.0036943)
Supplement: Methods S1 — (DOC) [file pone.0036943.s001.doc]

**Methods S1**

Complex demodulation

The analysis used in the present study was based on technique of Hayano et al [1]. In this analysis, signal X (t) is assumed to contain a frequency component which changes slowly around a specified center frequency. The process consists of four steps. First, shifting the frequency band around the frequency of interest to zero by multiplying the signal with a complex sinusoid at this center frequency:

where X(t) is the original signal as a function of time t, Y(t) is the complex signal after shifting the frequency band around the frequency of interest to zero as a function of time t, and f_0_ is a specified center frequency around which amplitude and phase (or frequency) deviations are calculated.

Second, the complex signal Y (t) is low-pass filtered so that only frequency components around zero remain. Third, the real and imaginary parts of the low-pass filtered signal are converted to polar form where the amplitude and phase as a function of time of the signal component at or near frequency f_0_ were obtained. Finally, the time series of the frequency deviations within the spectral region of interest is calculated as the slope of the phase of the phase vs. time curve. This last step is not presented in this study. Prior to complex demodulation the signals were padded with 100 points of mirror-imaged signal to avoid transients at the beginning of the computed results. Complex demodulation of SBP and IBI signals was performed at two frequency bands, as described.

SI -Reference

1. Hayano J, Taylor JA, Mukai S, Okada A, Watanabe Y, et al (1994). Assessment of frequency shifts in R-R interval variability and respiration with complex demodulation. J Appl Physiol 77:2879-2888.
